# Intravenous immune globulin suppresses angiogenesis in mice and humans

**DOI:** 10.1038/sigtrans.2015.2

**Published:** 2016-01-28

**Authors:** Reo Yasuma, Valeria Cicatiello, Takeshi Mizutani, Laura Tudisco, Younghee Kim, Valeria Tarallo, Sasha Bogdanovich, Yoshio Hirano, Nagaraj Kerur, Shengjian Li, Tetsuhiro Yasuma, Benjamin J Fowler, Charles B Wright, Ivana Apicella, Adelaide Greco, Arturo Brunetti, Balamurali K Ambati, Sevim Barbasso Helmers, Ingrid E Lundberg, Ondrej Viklicky, Jeanette HW Leusen, J Sjef Verbeek, Bradley D Gelfand, Ana Bastos-Carvalho, Sandro De Falco, Jayakrishna Ambati

**Affiliations:** 1 Department of Ophthalmology and Visual Sciences, University of Kentucky, Lexington, KY, USA; 2 Department of Ophthalmology, Nagoya University Graduate School of Medicine, Nagoya, Japan; 3 Angiogenesis Lab, Institute of Genetics and Biophysics—CNR, Naples, Italy; 4 Bio-Ker, MultiMedica Group, Naples, Italy; 5 Department of Physiology, University of Kentucky, Lexington, KY, USA; 6 Department of Advanced Biomedical Sciences, University of Naples ‘Federico II’, Naples, Italy; 7 CEINGE—Biotecnologie Avanzate, s.c.ar.l., Naples, Italy; 8 Department of Ophthalmology and Visual Sciences, Moran Eye Center, University of Utah School of Medicine, Salt Lake City, UT, USA; 9 Department of Ophthalmology, Veterans Affairs Salt Lake City Healthcare System, Salt Lake City, UT, USA; 10 Rheumatology Unit, Department of Medicine, Karolinska Institutet, Karolinska University Hospital, Stockholm, Sweden; 11 Department of Nephrology, Institute for Clinical and Experimental Medicine, Prague 4, Czech Republic; 12 Immunotherapy Laboratory, Laboratory for Translational Immunology, University Medical Center Utrecht, Utrecht, The Netherlands; 13 Department of Human Genetics, Leiden University Medical Center, Leiden, The Netherlands; 14 Department of Biomedical Engineering, University of Kentucky, Lexington, KY, USA; 15 Department of Microbiology, Immunology, and Molecular Genetics, University of Kentucky, Lexington, KY, USA; 16 IRCCS MultiMedica, Milano, Italy

## Abstract

Human intravenous immune globulin (IVIg), a purified IgG fraction composed of ~60% IgG1 and obtained from the pooled plasma of thousands of donors, is clinically used for a wide range of diseases. The biological actions of IVIg are incompletely understood and have been attributed both to the polyclonal antibodies therein and also to their IgG (IgG) Fc regions. Recently, we demonstrated that multiple therapeutic human IgG1 antibodies suppress angiogenesis in a target-independent manner via FcγRI, a high-affinity receptor for IgG1. Here we show that IVIg possesses similar anti-angiogenic activity and inhibited blood vessel growth in five different mouse models of prevalent human diseases, namely, neovascular age-related macular degeneration, corneal neovascularization, colorectal cancer, fibrosarcoma and peripheral arterial ischemic disease. Angioinhibition was mediated by the Fc region of IVIg, required FcγRI and had similar potency in transgenic mice expressing human FcγRs. Finally, IVIg therapy administered to humans for the treatment of inflammatory or autoimmune diseases reduced kidney and muscle blood vessel densities. These data place IVIg, an agent approved by the US Food and Drug Administration, as a novel angioinhibitory drug in doses that are currently administered in the clinical setting. In addition, they raise the possibility of an unintended effect of IVIg on blood vessels.

## Introduction

Human intravenous immune globulin (IVIg) is a biological product obtained by pooling polyclonal IgG from thousands of healthy donors. It is approved for the treatment of numerous primary immunodeficiencies.^1^ It is also widely used in an ‘off-label’ manner to treat a wide range of dermatological, neurological, inflammatory and transplantation-related diseases. The biological actions of IVIg have been attributed both to the polyclonal specificities of the antibodies therein^2^ and to immunomodulatory or anti-inflammatory effects driven by their IgG Fc regions.^3,4^ In a companion paper, we demonstrate that therapeutic human IgG1 antibodies can suppress angiogenesis in a target-independent manner via FcγRI,^5^ a high-affinity receptor for IgG1.^6–8^ Therefore, we tested whether IVIg, which is composed of ~60% IgG1, also possessed similar anti-angiogenic properties.

## Materials and methods

### Animals

All animal experiments were in accordance with the guidelines of the relevant institutional authorities. Male mice, aged 4–8 weeks, were randomized 1:1 to treatment with active drug versus inactive drug or control treatments.

### Drug injections

For systemic administration in corneal, choroid and hind limb angiogenesis experiments, human IVIg (0.017–2 g/kg/dose; Gammagard, Baxter (Deerfield, IL, USA) or Privigen, CSL Behring (King of Prussia, PA, USA)) or PBS was injected into the tail vein immediately after injury and 3 days later. In tumor experiments, IVIg was injected twice a week. For intravitreous administration in choroidal angiogenesis experiments, human IVIg (40 μg, 1 μl) or PBS was administered into the vitreous humor of mice using a 33-gauge double-caliber needle (Ito Corporation, Fuji, Japan) once, immediately after laser injury, as previously described.^9^ *FCGRIA* or *Luc* small interfering RNAs (2 μg, 1 μl) was administered into the vitreous 1 day before intravitreous human IVIg administration and laser treatment.

### Corneal angiogenesis

Nylon sutures (Mani, Utsunomiya, Japan) were placed into the corneal stroma of mice, and on day 10 after injury, we calculated the mean percentage CD31^+^Lyve1^−^ blood vessel areas for corneal flat mounts with ImageJ (US National Institutes of Health, Bethesda, MD, USA) as previously reported.^10,11^

### Choroidal angiogenesis

Laser photocoagulation (OcuLight GL, IRIDEX, Mountain View, CA, USA) was performed on both eyes of mice to induce neovascularization, and on day 7 after injury, choroidal angiogenesis volumes were measured by scanning laser confocal microscopy (TCS SP5, Leica, Wetzlar, Germany) as previously reported with 0.7% fluorescein isothiocyanate-conjugated Isolectin B4 (Vector Laboratories, Burlingame, CA, USA).^12^

### Hind limb ischemia angiogenesis

Unilateral proximal femoral artery ligation was performed as previously described,^13^ and on day 7 after surgery, both anterior and posterior muscles from ischemic and non-ischemic hind limbs were harvested and processed for immunohistochemical analysis for vessel quantification. Color laser Doppler analysis was also performed using a dedicated Laser Doppler Perfusion Imaging System (PeriScan PIM II System, Perimed AB, Järfälla, Sweden).

### Tumor experiments

HCT-116 colon carcinoma cells for xenograft tumors and T241 fibrosarcoma cells for syngenic tumors were injected s.c. into the right flank of CD1 nude athymic mice or C57Bl/6J and *Fcgr1*^−/−^ mice, respectively. Tumor growth was monitored by measuring the shortest (*d*) and the longest (*D*) diameters using a caliper. The volume (TV) was calculated according to the formula: TV (mm^3^)=*d*^2^×*D*/2.

### Statistical analyses

Choroidal angiogenesis volumes per laser lesion were compared by hierarchical logistic regression using repeated measures analysis as previously described.^14^ Differences in pre- and post-treatment blood vessel densities in human tissue biopsies were compared by two-tailed paired Student *t*-test, with mean and 95% confidence interval values reported. For other comparisons, we used the Mann–Whitney *U*-test with Bonferroni correction for statistical comparison of multiple variables. Results are expressed as mean±s.e.m. Type-I error not exceeding 0.05 was deemed significant.

## Results

**Figure 1:**
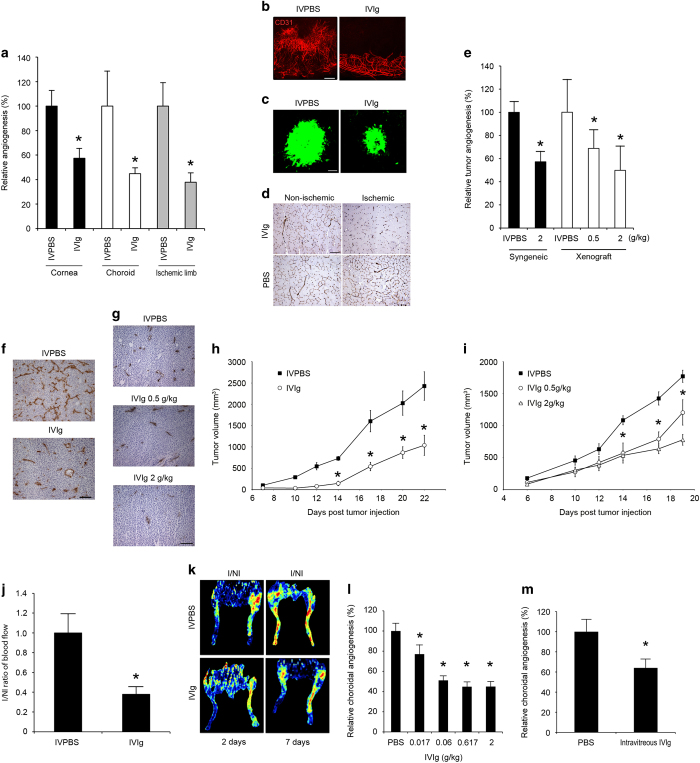
IVIg inhibited angiogenesis in five mouse models. (**a**) IVIg decreased choroidal angiogenesis, corneal angiogenesis and ischemic hind limb angiogenesis in wild-type mice. Choroidal angiogenesis volume was measured 7 days after laser injury and normalized to PBS treatment (IV PBS); *n*=8. Corneal area of angiogenesis was measured 10 days after suture injury and normalized to PBS group; *n*=16. Quantification of muscle CD31 immunolocalization was done 7 days after injury; *n*=7. (**b**) Representative photos of corneal flat mounts showing reduced growth of blood vessels (CD31+, red) in eyes treated with IVIg, but not in eyes treated with IV PBS. Scale bar, 500 μm. (**c**) Representative choroidal angiogenesis lesions (endothelial cells stained in green) show reduced choroidal angiogenesis in mice treated with IVIg but not in mice treated with IV PBS. Scale bar, 50 μm. (**d**) Representative images of muscle CD31 immunolocalization in the different treatment groups of the hind limb ischemia model. Scale bar, 100 μm. (**e**–**g**) IVIg decreased syngeneic tumor angiogenesis in wild-type mice and xenograft tumor angiogenesis in nude mice, as seen in (**e**) quantification of tumor CD31 immunolocalization (*n*=7), and representative histology images of syngenic (**f**) and xenograft (**g**) tumor tissue (CD31+, brown). The angioinhibitory effect was dose-dependent in the xenograft tumor model. IVIg doses ranged from 0.5 to 2 g/kg, delivered twice weekly. Scale bar, 100 μm. (**h**, **i**) IVIg reduced tumor growth in the syngeneic model (**h**) and in the xenograft model (**i**). In the xenograft model, the reduction in tumor volume was dose-dependent. IVIg doses ranged from 0.5 to 2 g/kg, delivered twice weekly; *n*=7. (**j**, **k**) Treatment of ischemic hind limb with IVIg in wild-type mice suppressed muscle revascularization and decreased blood vessel perfusion, as seen in (**j**) measured blood flow in the ischemic limbs, normalized to the contralateral non-ischemic limbs, 7 days after surgery and (**k**) representative laser Doppler perfusion images. (**l**) Systemic IVIg suppressed laser injury-induced choroidal angiogenesis volume in a dose-dependent manner. IVIg doses ranged from 0.017 to 2 g/kg, delivered twice (on the day of injury and 3 days later); *n*=6–8. (**m**) Local intravitreous injection of IVIg (40 μg in 1 μl) suppressed choroidal angiogenesis in wild-type mice; *n*=12. Results are means±s.e.m. *P<0.05 compared with PBS or IV PBS.

We tested the effect of IVIg in the following five different mouse models of angiogenesis: laser-induced choroidal angiogenesis, a model of neovascular age-related macular degeneration (AMD),^15,16^ suture-induced corneal angiogenesis,^10,11^ hind limb ischemia induced by femoral artery ligation^11,13^ and a syngeneic mouse fibrosarcoma tumor model,^17^ all in wild-type mice, as well as a human xenograft model of colorectal carcinoma in nude mice.^18^ IVIg inhibited angiogenesis in all five models (Figures 1a–g). In addition, and consistent with the concept that reduction of tumor vascularization induces tumor growth inhibition,^19–21^ tumor volume was reduced in IVIg-treated mice in both tumor models (Figures 1h and i). Furthermore, IVIg reduced muscle vascular reperfusion as measured by laser Doppler imaging (Figures 1j and k). IVIg also reduced infiltration of F4/80+ macrophages, which have a key role in multiple models of angiogenesis, into the ischemic hind limb of wild-type mice (Supplementary Figure 1).

High-dose IVIg (2 g/kg of body weight) is commonly administered for the treatment of autoimmune or inflammatory diseases.^3^ We confirmed that the anti-angiogenic effect of IVIg was dose-dependent and occurred with clinically relevant doses (0.017–2 g/kg of body weight) in wild-type mice in both the colorectal cancer and choroidal angiogenesis models (Figures 1e, g and l). Similarly, the observed reduction in tumor volume was also dose-dependent (Figure 1i).

Because local therapy in the form of intraocular injections is widely used in ophthalmic disorders, we next sought to determine whether IVIg also inhibited angiogenesis when delivered locally. We tested the effect of IVIg on choroidal angiogenesis when administered by intravitreous injection. IVIg delivered by this local route decreased angiogenesis as effectively as by IV administration (Figure 1m; compare with Figure 1a).

**Figure 2:**
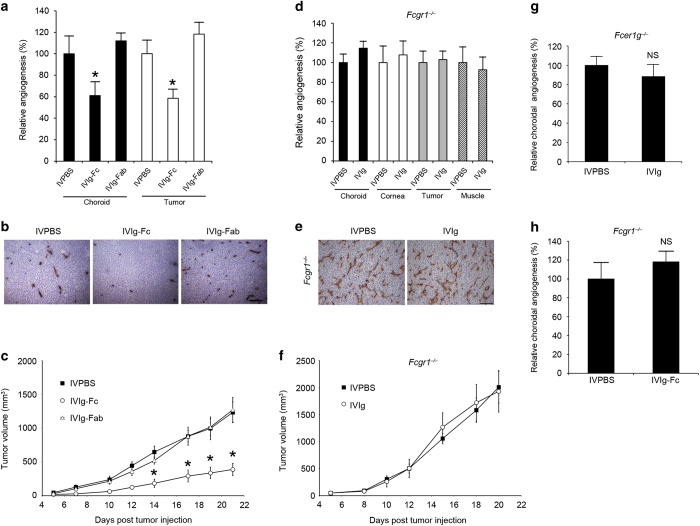
IVIg inhibited mouse angiogenesis via its Fc fragment and FcγRI engagement. (**a**, **b**) The Fc fragment of IVIg (IVIg-Fc), but not the Fab fragment (IVIg-Fab), inhibited choroidal angiogenesis in wild-type mice and xenograft tumoral angiogenesis in nude mice, as seen in (**a**) quantification of choroidal angiogenesis volume (*n*=4–8) and tumor CD31 immunolocalization (*n*=7) compared with PBS group (IV PBS), and (**b**) representative histology images of tumor tissue (CD31+, brown). Scale bar, 100 μm. (**c**) IVIg-Fc, but not IVIg-Fab, suppressed xenograft tumor growth, as compared with PBS. (**d**) IVIg did not inhibit choroidal (*n*=6–8), corneal (*n*=8) syngeneic tumor (*n*=7) or muscle (*n*=8) angiogenesis in *Fcgr1*^−/−^ mice, which lack FcγRI. No significant difference between groups. (**e**), Representative histology images of syngeneic tumor tissue in *Fcgr1*^−/−^ mice (CD31+, brown) treated with either IVIg or IV PBS. Scale bar, 100 μm. (**f**) IVIg did not suppress tumor growth in *Fcgr1*^−/−^ mice, as compared with IV PBS. *n*=7. (**g**) IVIg did not inhibit choroidal angiogenesis in *Fcer1g*^−/−^ mice, which lack functional signaling of activating FcγRs; *n*=6. (**h**) IVIg-Fc did not inhibit choroidal angiogenesis in *Fcgr1*^−/−^ mice. *n*=6. Results are means±s.e.m. *P<0.05 compared with IV PBS.

IVIg contains thousands of polyvalent antibodies, and it is possible that the Fab regions of some of them could target angiogenic molecules. Indeed, certain anti-inflammatory actions of IVIg have been attributed to the presence of specific antigen-targeting antibodies.^2^ To test which region of IVIg was responsible for its angioinhibitory effect, we treated mice with papain-derived Fc (IVIg-Fc) or Fab (IVIg-Fab) fragments of IVIg (Supplementary Figure 2). Consistent with the idea that IgG-mediated angioinhibition is not because of the specific antigen–antibody targeting of angiogenic molecules,^5^ systemic administration of IVIg-Fc inhibited choroidal and tumor angiogenesis in wild-type mice, whereas administration of IVIg-Fab by the same route did not do so (Figures 2a and b). As we had observed previously with full-length IVIg-Fc also inhibited xenograft tumor growth, whereas IVIg-Fab had no effect (Figure 2c).

IVIg has been reported to suppress inflammation in mice both via activating^22^ and inhibitory^23^ receptors for the Fc region of IgGs, the FcγRs. We recently showed that multiple human IgG1 antibodies and their Fc fragments suppressed angiogenesis via the activating FcγRI, and this anti-angiogenic effect was abolished in mice deficient in this receptor.^5^ Consistent with those findings, IVIg did not suppress angiogenesis in *Fcgr1*^−/−^ mice, which do not express FcγRI (Figures 2d and e), or in *Fcer1g*^−/−^ mice, which do not express the γ-chain of Fc receptors, therefore lacking signaling for all activating FcγRs (Figure 2g). In contrast, IVIg did suppress angiogenesis in *Fcgr2b*^−/−^ mice, which do not express the inhibitory FcγRII (Supplementary Figure 3). Tumor growth was also not abrogated by IVIg in *Fcgr1*^−/−^ mice (Figure 2f). Furthermore, and consistent with the hypothesis that IVIg inhibits blood vessel growth via interaction of its Fc fragments with FcγRI, papain-cleaved IVIg-Fc also did not reduce angiogenesis in *Fcgr1*^−/−^ mice (Figure 2h).

**Figure 3:**
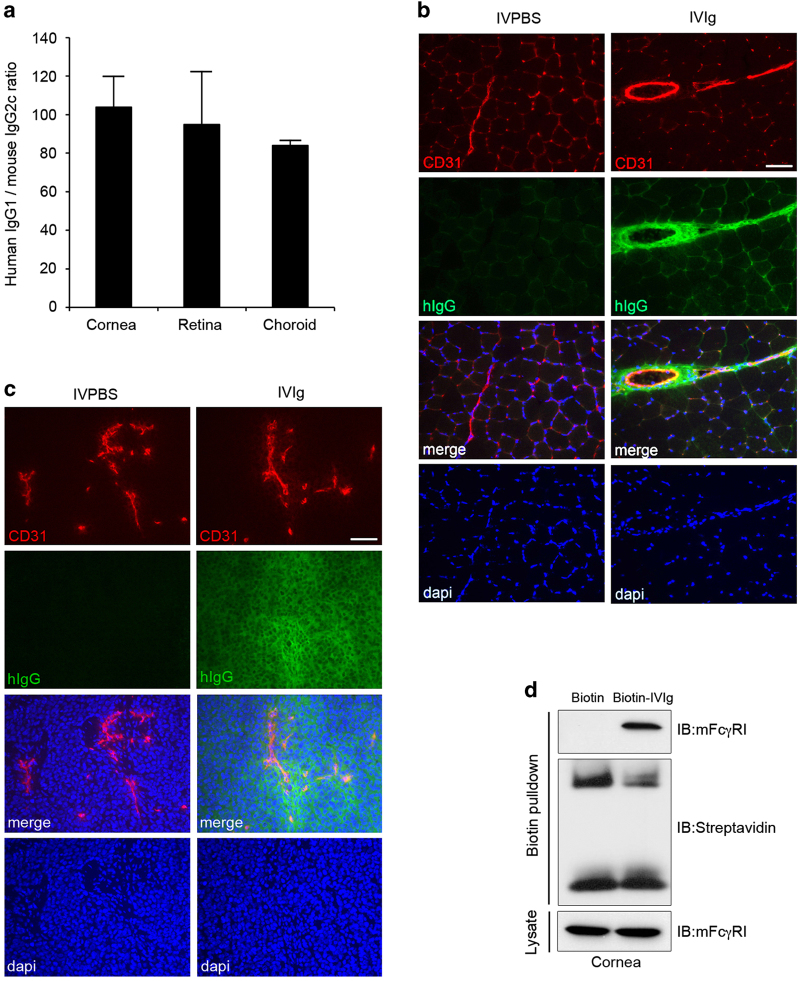
IVIg is present in tissue where angioinhibition occurs and binds mouse FcγRI (mFcγRI). (**a**) IVIg injected IV was present in corneal, retinal and choroidal tissues, and its levels exceeded those of endogenous mouse IgG2c, as assessed by human IgG1 enzyme-linked immunosorbent assay (ELISA) and mouse IgG2c ELISA. Mice were subjected to corneal suture placement or choroidal laser injury, and IVIg was injected in the same scheme used for *in vivo* angiogenesis experiments (2 g/kg, on days 1 and 3). Tissue was harvested and processed for ELISA on day 3. (**b**) Immunostaining of muscle tissue in wild-type mice was positive for human IgG after systemic administration of IVIg. Scale bar, 100 μm. (**c**) Immunostaining of tumor tissue from colon carcinoma xenografts in nude mice was positive for human IgG after systemic administration of IVIg. Scale bar, 100 μm. (**d**) Pull-down assay revealed the presence of IVIg in corneal tissue following suture injury. After systemic administration, biotinylated IVIg was pulled down from the corneal tissue lysate and the eluted proteins were blotted for mFcγRI.

To confirm the *in vivo* existence of IVIg-FcγRI engagement in the angiosuppressive process, we assessed the presence of IVIg in the injury sites of the different mouse models after its IV administration by multiple strategies. First, we assessed the extravascular levels of human IgG (corresponding to the injected IVIg of human origin) by enzyme-linked immunosorbent assay in the corneal, retinal and choroidal tissues, and verified that they greatly exceeded those of endogenous mouse IgG2c (Figure 3a), the IgG isotype of C57BL/6J mice that binds mFcγRI with high affinity.^24^ Second, using immunostaining in the hind limb ischemia and xenograft colon carcinoma models, we visualized human IgG in the extra-fiber space of muscle and also in the tumor stroma after administering systemic IVIg (Figures 3b and c). Finally, using a pull-down assay, we determined that biotinylated IVIg injected IV into wild-type mice co-precipitated with FcγRI in their corneas after suture injury (Figure 3d). Collectively, these data suggest that, similarly to human IgG1, IVIg interacts with FcγRI *in vivo* and suppresses angiogenesis in mice via this receptor. Igs bind not only to FcγRs but also to C1q, and some effects of IVIg have been attributed to complement activation.^25^ However, IVIg retained its anti-angiogenic activity in *C1qa*^−/−^ mice (Supplementary Figure 4), suggesting that complement activation is not required for this function of IVIg.

**Figure 4:**
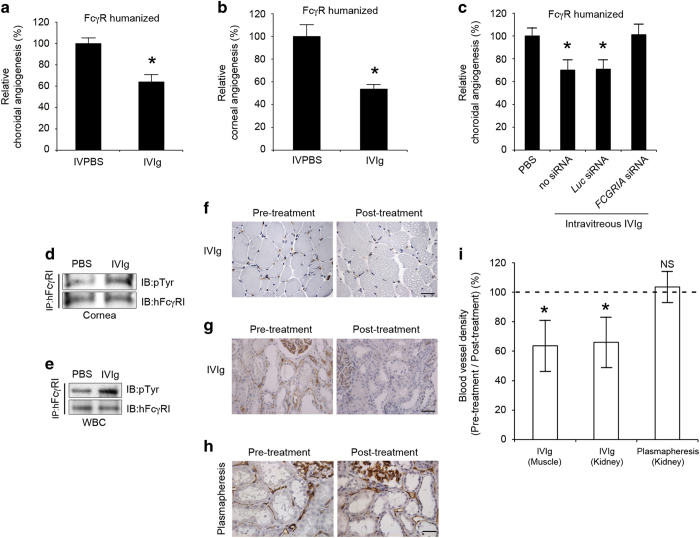
IVIg is angioinhibitory in FcγR humanized mice and in human patients. (**a**) IVIg inhibited choroidal angiogenesis in FcγR humanized mice, which express the human isoforms of FcγRs and lack the mouse isoforms of these receptors, as compared with IV PBS; *n*=6. (**b**) IVIg inhibited corneal angiogenesis in FcγR humanized mice; *n*=6. *P<0.05 compared with IV PBS (**a**, **b**). (**c**) Intravitreous IVIg did not inhibit choroidal angiogenesis in FcγR humanized mice when co-administered with an intravitreous small interfering RNA targeting *FCGR1A*, the human gene encoding FcγRI; *n*=12–14. *P<0.05 compared with PBS and *FCGR1A* small interfering RNA. (**d**, **e**) IVIg induced phosphorylation of FcγRI in humanized mice. Mice were subjected to corneal suture placement and treatment with IVIg or IV PBS, and (**d**) cornea or (**e**) white blood cell lysates were immunoprecipitated with anti-FcγRI antibodies and were immunoblotted with the indicated antibodies. (**f**–**h**) Representative immunohistochemistry images of biopsies of (**f**) muscle or (**g**) kidney from patients treated with IVIg, or (**h**) kidney from patients treated with plasmapheresis, showing tissue density of blood vessels (CD31+, brown) is reduced after treatment with IVIg, but not after plasmapheresis. Scale bars, 50 μm. (**i**) Mean changes in blood vessel density (post- versus pre-treatment). Error bars indicate 95% confidence intervals. *P=0.002 (*n*=8, muscle), *P=0.006 (*n*=10, kidney, IVIg-treated patients), not significant (NS; *n*=3, kidney, plasmapheresis-treated patients) comparing post- versus pre-treatment, two-tailed paired Student *t*-test.

Although human IgG1 binds both mouse and human FcγRI,^26^ mouse and human FcγRs have species-specific structural diversity and cellular expression patterns.^27^ Therefore, we sought to determine whether the angioinhibitory effects of IVIg in mice also could be observed in the setting of human FcγRs. We first studied the transgenic FcγR humanized mouse, which expresses the entire human FcγR family, under the control of their human regulatory elements, on a genetic background lacking all mouse FcγRs.^28^ Consistent with our observation,^5^ as well as those of others,^29^ that human IgG1 binds human FcγRI *in vivo*, and with the notion that IVIg mediates angioinhibition via FcγRI, IVIg reduced choroidal and corneal angiogenesis in FcγR humanized mice as efficiently as in wild-type mice (Figures 4a and b). Further, concomitant administration of IVIg with a small interfering RNA targeting *FCGRIA* (the gene encoding human FcγRI) abrogated this angioinhibitory effect in FcγR humanized mice (Figure 4c). In this humanized model, IVIg induced phosphorylation of FcγRI, both in the corneas and in the white blood cells of mice (Figures 4d and e), thereby strengthening the notion that IVIg induces signaling via this receptor.

We then examined renal or muscle biopsies of human patients obtained before and after treatment with IVIg for renal transplant rejection^30^ or inflammatory myopathies,^31^ respectively (Supplementary Table). Strikingly, biopsies of patients obtained after receiving IVIg displayed reduced blood vessel density in the kidneys or muscles; however, there was no reduction in blood vessel density in the kidneys of patients that underwent plasmapheresis as an alternative therapy to IVIg (Figures 4f–i). These data, derived from genetically diverse patient populations in different countries, are a confirmation that in humans clinical doses of IVIg can modulate blood vessel density. Although these findings are consistent with our data on suppression of angiogenesis by IVIg via FcγRI in numerous mouse models, we cannot conclude that the reduced blood vessel density in patients treated with IVIg is mediated by FcγRI, as it is possible that other effects of IVIg might be responsible for this action in humans.

## Discussion

Here we present the discovery that IVIg suppresses blood vessel growth via Fc-FcγRI engagement. IVIg inhibited ocular, muscle and tumor angiogenesis, demonstrating a broad angioinhibitory effect on varying blood vessel types and in diverse tissue environments. Further, the human data demonstrating that IVIg therapy modulates angiogenesis when administered at a dose routinely used in the clinical setting indicates that IVIg-induced angioinhibition also occurs in humans. These data go hand-in-hand with our recent findings that multiple therapeutic human monoclonal IgG1 antibodies, as a class, possess the same angioinhibitory effect via FcγRI.^5^

Treatment with IVIg has been reported to be anti-metastatic both in animal models^32^ and in humans.^32,33^ Our demonstration that IVIg concomitantly decreases blood vessel density and tumor growth suggests that angioinhibition might be an important determinant in its effect on decreasing tumor progression and invasion. We recently showed that hIgG1-mediated angioinhibition is associated with decreased macrophage migration and infiltration of angiogenic sites via FcγRI engagement.^5^ We have demonstrated a similar inhibitory effect on macrophage migration by IVIg in ischemia muscle. Because infiltration of tumors by tumor-associated macrophages is associated with increased cancer vascular grade and progression,^34^ such a mechanism of action of IVIg might contribute to the decrease in tumor volume we observed.

The neovascular form of AMD, the leading disease responsible for blindness in the elderly in industrialized countries,^35^ is caused by choroidal angiogenesis in the macula, the central region of the retina responsible for high-acuity vision. Currently, the standard treatment for this disease is monthly intraocular injections of anti-VEGFA agents such as bevacizumab, ranibizumab or aflibercept. These drugs are currently responsible for roughly one-sixth of the Medicare Part B fee-for-service program expenses.^36–38^ In this work we demonstrate that, in the mouse model of neovascular AMD we used, 40 μg of intravitreous IVIg inhibited choroidal angiogenesis as effectively as anti-VEGFA antibodies do.^5^ Translated to the human eye vitreous volume, this would correspond to an approximate dose of 22 mg (which is contained in 220 μl of the commercially available IVIg preparations at 100 mg/ml), making it substantially more affordable than current US food and drug administration-approved therapies. Further, intravitreous IVIg has been previously shown to not induce retinal inflammation in a rabbit model, in doses far higher than the ones used in our studies,^39^ suggesting that it is likely to be safe for use in human eye diseases.

Our data also raise the possibility that IVIg might induce undesirable effects on the blood vasculature in other diseases and should prompt monitoring for such effects. The potential functional consequences of such vascular disturbances remain to be determined. Among the innumerable diseases for which IVIg is used in an ‘off-label’ manner is Alzheimer’s disease. Although it has been postulated that IVIg clears β-amyloid deposits,^40^ a phase 3 clinical trial recently reported that IVIg did not provide benefit in Alzheimer’s disease. Whether suppression of angiogenesis by IVIg via FcγRI might have contributed to this absence of therapeutic benefit warrants further study. Likewise, it would be worthwhile exploring whether possibly detrimental FcγRI-mediated angioinhibition had a role in the failures of several monoclonal antibody therapies in clinical trials in Alzheimer’s disease and diabetes.

Our studies put forward a novel role for IVIg as an angiosuppressive drug, potentially applicable in multiple human diseases. IVIg may be ripe for rapid repurposing as a systemic angioinhibitory agent and in the near future as an intraocular inexpensive therapy for multiple neovascular blinding diseases, such as AMD, proliferative diabetic retinopathy or retinopathy of prematurity. We also propose that, in view of our data, systemic use of IVIg should be accompanied by monitoring of adverse effects in blood vessels, particularly in patients at risk for vascular diseases.
