# Supplementary material for: Intravenous immune globulin suppresses angiogenesis in mice and humans
Source: Signal Transduct Target Ther. 2016 Jan 28;1:15002–. doi: 10.1038/sigtrans.2015.2 (PMC4768485; doi:10.1038/sigtrans.2015.2)
Supplement: Supplementary Information [file sigtrans20152-s1.pdf]

Supplementary Figure 1

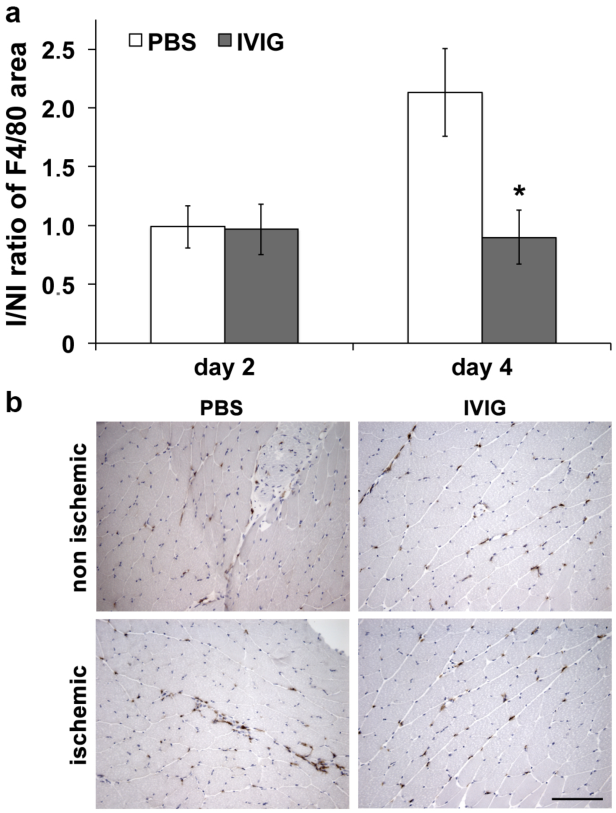

Supplementary Figure 1. Reduced number of F4/80+ (brown) macrophages in muscle of ischemic hind limbs treated with IVIg, as compared with IV PBS treatment, as seen in representative pictures (**b**) and macrophage quantification (**a**), 4 days after surgery. Scale bar, 100 μm (**b**). Results are means ± SEM (n = 6–8). * $P < 0.05$ compared with IV PBS.

Supplementary Figure 2

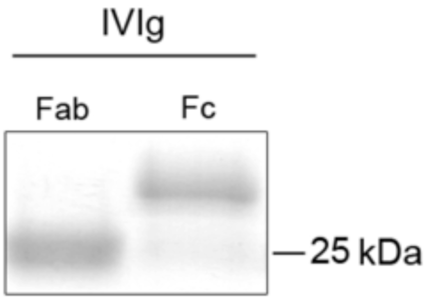

Supplementary Figure 2. Coomassie stained SDS-page gel with complete separation of Fab and Fc fragments of full-length IVIg after papain digestion.

Supplementary Figure 3

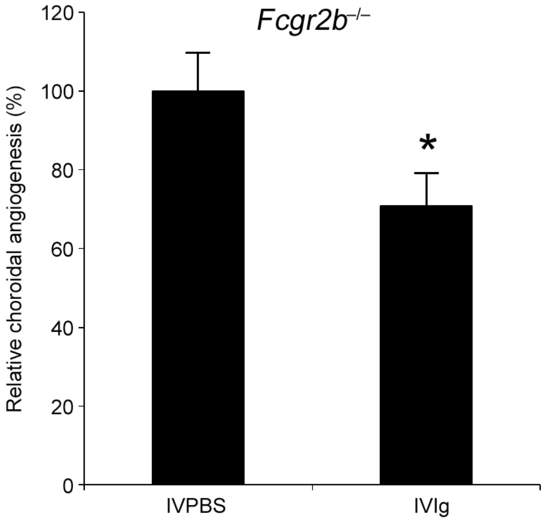

Supplementary Figure 3. Intravenous IVIg decreased choroidal angiogenesis in *Fcgr2b*$^{-/-}$ mice mice. Choroidal angiogenesis volume was measured 7 days after laser injury and normalized to PBS treatment (IVPBS, intravenous PBS). n = 6. Results are means ± SEM. * $P < 0.05$ compared with IVPBS.

Supplementary Figure 4

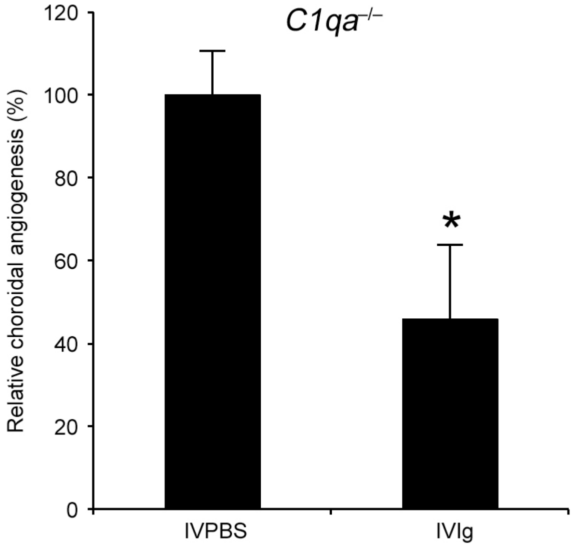

Supplementary Figure 4. Intravenous IVIg decreased choroidal angiogenesis in *C1qa*$^{-/-}$ mice mice. Choroidal angiogenesis volume was measured 7 days after laser injury and normalized to PBS treatment (IVPBS, intravenous PBS). n = 6. Results are means ± SEM. * $P < 0.05$ compared with IVPBS.

Supplementary Table

IVIg – Muscle

Donor	Age (Years)	Gender	IVIg infusions (#)
1	54	F	3
2	55	F	3
3	52	M	3
4	64	F	3
5	71	F	3
6	63	F	3
7	67	M	3
8	52	F	3

IVIg – Kidney

Donor	Age (Years)	Gender	IVIg infusions (#)
1	73	F	5
2	59	F	5
3	57	M	5
4	47	M	5
5	39	F	5
6	54	F	11
7	44	M	7
8	40	F	7
9	38	M	12

10	64	M	7

Plasmapheresis – Kidney

**Donor**	**Age (Years)**	**Gender**
1	66	M
2	24	M
3	55	F

Methods

Animals. All animal experiments were in accordance with the guidelines of the University of Kentucky Institutional Animal Care and Use Committee, and the Association for Research in Vision and Ophthalmology (ARVO) Animal Statement for the Use of Animals in Ophthalmic and Vision Research. The hind limb ischemia and tumor experimental procedures were in accordance with European Directives no. 2010/63/EU and Italian D.L. 26/2014 and approved by the veterinarian of the Institute of Genetics and Biophysics. C57BL/6J and *Fcgr2b*$^{-/-}$ mice were purchased from The Jackson Laboratory, and CD1 nude athymic mice from Charles River. *C1qa*$^{-/-}$, FcγR humanized,[1] *Fcgr1*$^{-/-}$, and *Fcer1g*$^{-/-}$ mice were obtained from M. Botto (via L.E. Nagy), J.V. Ravetch, J.S. Verbeek, and J.H.W. Leusen respectively. Male mice, aged 4–8 weeks, were randomized 1:1 to treatment with active drug versus inactive drug or control treatments. For all procedures, anesthesia was performed by intraperitoneal injection of 100 mg/kg ketamine hydrochloride (Ft. Dodge Animal Health) and 10 mg/kg xylazine (Phoenix Scientific). Pupils were dilated with topical tropicamide (1%; Alcon Laboratories). Operator were masked to treatment groups while conducting analyses.

Drug injections. For systemic administration in corneal, choroid and hind limb angiogenesis experiments, human IVIg (0.017–2 g/kg/dose; Gammagard™, Baxter or Privigen™, CSL Behring) or PBS was injected into the tail vein immediately after injury and 3 days later. In tumor experiments, IVIg was injected twice a week. For intravitreous administration in choroidal angiogenesis experiments, human IVIg (40 μg, 1ul) or PBS was administered into the vitreous humor of mice using a 33-gauge double-calibre needle (Ito Corporation) once, immediately after laser injury, as previously described.[2] *FCGRIA* or *Luc* siRNAs (2 μg, 1 μl) was administered into

the vitreous 1 day prior to intravitreous human IVIg administration and laser treatment.

Intravenous Immune globulin Fab/Fc fragmentation. 15 ml of IVIg solution (100 mg/ml) was mixed with 13.5 ml Digestion buffer (41 mM EDTA, 60 mM L-Cystein in PBS) and 1.5 ml Papain (blurry solution from Sigma, 24 mg/ml). The mixture was incubated at 37 °C for 2 h, and 80 mg of iodoacetamide was added to stop the reaction. HiPrep 26/60 Sephacryl S-300 HR Column (GE Healthcare) was then used to separate the undigested IVIg and Fab/Fc. The corresponding Fab/Fc mixture was firstly separated using hooked Protein G columns (HiTrap Protein G HP). The Fab/Fc mixture was eluted using Elution buffer (0.1M HCl-Glycine, pH 2.7). The elution was neutralized using 1M Tris-HCl pH 9.0 when collected. The eluted Fab/Fc mixture from Protein G columns was further separated using hooked Protein L columns (HiTrap Protein L HP), leaving Fc in the flow-through. The Fab fragment was eluted using Elution buffer (0.1M HCl-Glycine pH 2.7) and neutralized with 1M Tris-HCl pH 9.0. Fab fractions from Protein G and Protein L column were combined, thoroughly dialyzed against PBS and concentrated. Fc fraction from Protein L columns was thoroughly dialyzed against PBS and concentrated. The purified Fab and Fc of IVIg were kept at –80 °C. The Fab and Fc fractions were confirmed by SDS-PAGE and concentrated using a Vivaspin 20 centrifugal concentrator (10,000 Dalton molecular weight cutoff; Sartorius Stedim Biotech).

Corneal angiogenesis. Two interrupted 11–0 nylon sutures (Mani) were placed into the corneal stroma, midway between the central corneal apex and the limbus (approximately 1.25 mm from the limbus), of both eyes of mice as previously described.[3,4] On day 10 after injury, we calculated the mean percentage $CD31^{+}Lyve1^{-}$ blood vessel areas for corneal flat mounts with

ImageJ (US National Institutes of Health) as previously reported.[3,4] Eyes were excluded in masked fashion from analyses (<5% incidence) if anterior chamber hemorrhage, suture loosening, or cataract formation occurred at any time during the study period.

Choroidal angiogenesis. Laser photocoagulation (OcuLight GL, IRIDEX) was performed on both eyes of mice to induce CNV as previously described.[5] Choroidal angiogenesis volumes were measured by scanning laser confocal microscopy (TCS SP5, Leica) 7 days after injury as previously reported with 0.7% FITC-conjugated Isolectin B4 (Vector).[5] Laser lesions were excluded in masked fashion from analyses (5% incidence) if laser photocoagulation did not induce a bubble, if it induced hemorrhage, or if lesions became confluent with one another.

Hind limb ischemia angiogenesis. Mice were anesthetized before undergoing unilateral proximal femoral artery ligation. The right femoral artery was gently isolated, ligated and excised distal to the deep femoral artery and 0.5 cm proximal to the bifurcation in saphenous and popliteal arteries, as previously described.[6] The non-ischemic left limb underwent sham surgery without arterial ligation. On day 7 after surgery, both anterior and posterior muscles from ischemic and non-ischemic hind limbs were harvested and processed for immunohistochemical analysis for vessel quantification. Animals were excluded in masked fashion from analyses (0% incidence) if hemorrhagic death from improper ligation occurred.

Color laser Doppler analysis. Color laser Doppler analysis was performed 7 days after femoral artery ligation using a dedicated Laser Doppler Perfusion Imaging System (LDPI, PeriScan PIM II System, Perimed AB) with high resolution, in single mode. Hind limbs were depilated and

mice were placed on a heating plate at 37 °C. The distance between the scanner head and tissue surface was 8 cm. An area of 5 × 5 cm was sequentially scanned and blood flow 1 mm under the surface was measured. Color-coded images were recorded, and analyses were performed calculating the average perfusion of the right and left distal limb. Dark blue color implied low or absent perfusion whereas red implied maximal perfusion.

IHC analysis for monocyte-macrophage infiltrate. Five µm-thick muscle cryosections were incubated overnight at 4 °C with rat anti-mouse F4/80 (Serotec, Oxford, UK) antibody diluted 1:50. The staining procedure was continued using specific secondary biotinylated antibody (DAKO, Glostrup, Denmark). Slides were counterstained with hematoxylin. Images were recorded with a digital Leica DC480 camera (Milano, Italy). Densitometric analysis for F4/80 staining was performed with QwinPro software (Leica).

Tumor experiments. 3 x 10^6 HCT-116 colon carcinoma cells for xenograft tumors, and $5x10^5$ T241 fibrosarcoma cells for syngenic tumors, were injected subcutaneously into the right flank of CD1 nude athymic mice or C57Bl/6J and *Fcgr1*$^{-/-}$ mice, respectively, and maintained under pathogen-free conditions at IGB Animal House Facility. After 5-6 days, when tumors reached a volume between 50 and 100 mm^3, animals were randomly divided into groups (n = 7) and injected twice a week with: IVIg 0.5 g/kg, IVIg 2 g/kg, IVIG-Fc 0.66 g/kg, IVIG-Fab 1.33 g/kg or PBS as control. The tumor growth was monitored three times at week by measuring the shortest (d) and the longest (D) diameters using a caliper. The volume (TV) was calculated according to the formula: $TV(mm^3)= d^2 \times D/2$. For ethical reasons, mice were sacrificed when control tumors reached in average a volume between 1,500 and 2,000 mm^3. Explanted tumors

were immediately frozen for successive analyses. ARRIVE guidelines and the Basel declaration were considered when planning the experiments.

IVIg ELISA. C57BL/6J mice were treated with IVIg 2g/kg (on days 1 and 3) and subjected to corneal suture placement (according to the corneal angiogenesis protocol described above), or to laser-induced choroidal angiogenesis (according to the choroidal angiogenesis protocol described above). On day 3, after transcardial perfusion with PBS, corneas were harvested from mice subjected to corneal suture, and retinas and choroids were harvested from mice subjected to choroidal laser. Corneas were chopped into fine pieces on ice. Corneal and retinal samples were homogenized by sonication. Samples were spun at 14,000Å~g for 20 min at 4 °C and the supernatant was transferred to a clean tube. Tissue supernatant was used to perform protein assays. Human IgG1 (Abcam) ELISA, and mouse IgG2c ELISA (Bethyl), were performed in all samples according to the manufacturer's instructions. Measurements were performed using Synergy 4 Microplate Reader and Gene5 software (BioTec, Suffolk, United Kingdom).

FcγRI interaction. IVIg was biotinylated using the EZ-Link Sulfo-NHS-LC-Biotinylation Kit (Thermo Scientific) by following the manufacturer's instructions. After the reaction, by using the HABA biotin assay (Pierce Biotin Quantitation Kit; Thermo Scientific), it was found that each protein molecule had been modified by ~ 3 biotin molecules. Biotinylated IVIg (0.667 g/kg of body weight) was injected into the tail vein of wild-type mice and followed by suture injury. Two days later, corneas were excised and cell lysates were prepared by pooling 6 corneas in each group. Equal amounts of total corneal protein (400 ug) were subjected to "pull-down" with Dynabeads® M-280 Streptavidin (Life Technologies) for 2 h at RT and eluted with protein

sample buffer. Eluted proteins prepared in Laemmli buffer were resolved by SDS-PAGE on Novex® Tris-Glycine Gels (Invitrogen), and transferred onto Immun-Blot PVDF membranes (Bio-Rad). The transferred membranes were blocked for 1 h at RT and incubated with antibody against mouse FcγRI (1:1000; Santa Cruz) at 4 °C overnight. The immunoreactive bands were developed by enhanced chemiluminescence reaction. The membrane was stripped and reprobed with HRP-conjugated streptavidin (BioLegend).

Human Studies – Kidney. The Ethics Committee of the Institute for Clinical and Experimental Medicine, Prague approved the study protocol and all patients signed informed consent to participate in the study, which was conducted between January 2002 and December 2006.[7] Patients with antibody-mediated kidney graft rejection received treatment based on plasmapheresis and/or IVIg 0.5 g/kg. Renal biopsies were obtained under ultrasound guidance (Toshiba, Power Vision 6000) using a 14-gauge Tru-Cut needle (Uni-Cut Nadeln, Angiomed, Germany) within 24 h before the first plasmapheresis/IVIg treatment and follow-up biopsies at 21 days after the last plasmapheresis/IVIg treatment. Biopsies were fixed in formalin and paraffin-embedded, and cut on 4 μm thick sections for immunohistochemistry. Plasmapheresis was performed on a Prisma system (HOSPAL, GAMBRO DASCO, Italy) with a high-permeability capillary filter. Separated plasma was substituted by human 20% albumin Ringer-lactate solution. Polyvalent human lyophilized immunoglobulin produced from plasma (Endobulin, Baxter-Immuno, Germany) was used as IVIg.

Human Studies – Muscle. Patients with inflammatory myopathies at the Rheumatology Unit, Karolinska University Hospital, Solna provided informed consent to participate, and the local

ethics committee Nord, Stockholm, approved the study. The patients were given infusions during 2–5 days with 2 g/kg of IVIg, three times, at monthly intervals as previously described.[8] A baseline muscle biopsy was performed within 24 h before the first IVIg treatment was initiated and a repeat biopsy was performed 2 weeks after the last IVIg treatment. The biopsy specimens were mounted in an embedding medium, frozen in isopentane pre-cooled by dry ice and stored at –80°C. For each biopsy, 7 mm thick serial cryostat sections were mounted on gelatin-coated glass slide and stored at –80°C until stained.

Tissue immunohistochemistry. Paraffin sections were deparaffinized in xylol and rehydrated in graded alcohol series whereas the muscle cryosections were dried out and fixed in 4% paraformaldehyde. Endogenous peroxidase was inhibited using 3% H_2O_2 in methanol. The sections were washed and boiled 15 min in 10 m*M* sodium citrate buffer for epitope retrieval. No pretreatment was needed for D2-40 staining. Endogenous biotin was inhibited with a Vector Blocking kit (Vector Laboratories). For human tissue monoclonal anti-human CD31 (1:50, Dako) was used. Bound antibody was detected with biotin-conjugated secondary antibodies (Dako). Counterstaining was performed with hematoxylin. Imaging was performed in masked fashion. Analyses were performed on five optical fields for each sample. For mouse tumor tissue, immunohistochemical analyses were performed on 5-µm-thick cryosections incubated overnight at 4 °C with the rat anti-mouse PECAM-1 (anti-CD31; 1:1000; BD Pharmingen) The staining procedure was continued using specific secondary biotinylated antibodySlides were counterstained with hematoxylin. Images were recorded with a digital camera Leica DC480 (Milano, Italy). Densitometric analysis for CD31 was performed with QwinPro software (Leica). Analyses were performed on five optical fields for each tumor.

Statistical analyses. Choroidal angiogenesis volumes per laser lesion were compared by hierarchical logistic regression using repeated measures analysis as previously described.[9] Differences in pre-treatment and post-treatment blood vessel densities in human tissue biopsies were compared by two-tailed paired Student t test, with mean and 95% C.I. values reported. For other comparisons, we used the Mann-Whitney *U* test with Bonferroni correction for statistical comparison of multiple variables. Results are expressed as mean ± s.e.m. Type-I error not exceeding 0.05 was deemed significant.

References

1 Smith, P., DiLillo, D. J., Bournazos, S., Li, F. & Ravetch, J. V. Mouse model recapitulating human Fcgamma receptor structural and functional diversity. *Proceedings of the National Academy of Sciences of the United States of America* **109**, 6181-6186, doi:10.1073/pnas.1203954109 (2012).

2 Bogdanovich, S. *et al.* Human IgG1 antibodies suppress angiogenesis in a target-independent manner. *Signal Transduction and Targeted Therapy*, In press (2016).

3 Albuquerque, R. J. *et al.* Alternatively spliced vascular endothelial growth factor receptor-2 is an essential endogenous inhibitor of lymphatic vessel growth. *Nat. Med.* **15**, 1023-1030, doi:10.1038/nm.2018 (2009).

4 Cho, W. G. *et al.* Small interfering RNA-induced TLR3 activation inhibits blood and lymphatic vessel growth. *Proc. Natl. Acad. Sci. U. S. A.* **106**, 7137-7142, doi:10.1073/pnas.0812317106 (2009).

5 Takeda, A. *et al.* CCR3 is a target for age-related macular degeneration diagnosis and therapy. *Nature* **460**, 225-230, doi:10.1038/nature08151 (2009).

6 Couffinhal, T. *et al.* Mouse model of angiogenesis. *Am. J. Pathol.* **152**, 1667-1679 (1998).

7 Slatinska, J., Honsova, E., Burgelova, M., Slavcev, A. & Viklicky, O. Plasmapheresis and intravenous immunoglobulin in early antibody-mediated rejection of the renal allograft: a single-center experience. *Ther. Apher. Dial.* **13**, 108-112, doi:10.1111/j.1744-9987.2009.00664.x (2009).

8 Barbasso Helmers, S. *et al.* Limited effects of high-dose intravenous immunoglobulin (IVIG) treatment on molecular expression in muscle tissue of patients with inflammatory myopathies. *Ann. Rheum. Dis.* **66**, 1276-1283, doi:10.1136/ard.2006.058644 (2007).

9 Kleinman, M. E. *et al.* Sequence- and target-independent angiogenesis suppression by siRNA via TLR3. *Nature* **452**, 591-597, doi:10.1038/nature06765 (2008).
